# Pulsed‐field ablation to isolate common inferior pulmonary veins in a patient with recurrent atrial fibrillation

**DOI:** 10.1002/joa3.70058

**Published:** 2025-04-09

**Authors:** Machiko Miyoshi, Kanae Hasegawa, Masato Shimada, Hiroshi Tada

**Affiliations:** ^1^ Department of Cardiovascular Medicine, Faculty of Medical Sciences University of Fukui Fukui Japan; ^2^ Department of Radiology University of Fukui Hospital Fukui Japan

**Keywords:** atrial fibrillation, catheter ablation, common trunk, pulmonary vein, pulsed‐field ablation

## Abstract

This is the first report of a successful isolation of the common trunk of inferior pulmonary veins (PV) using pulsed‐field ablation (PFA). PFA is easier and more reliable than conventional ablation methods for ablation in patients with PV anomalies and/or a small left atrium and for extensive ablation such as box isolation.

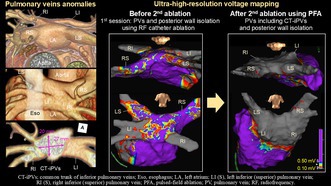

A 76‐year‐old man was referred to our hospital for the recurrence of paroxysmal atrial fibrillation (AF). Two years earlier, the patient had undergone radiofrequency catheter ablation for a left atrial (LA) posterior wall isolation in addition to pulmonary vein (PV) isolation at another hospital. Three‐dimensional reconstruction computed tomography demonstrated a common trunk of the inferior PVs (CT‐iPVs; Figure 1). Ultra‐high‐resolution voltage mapping during coronary sinus pacing with an Advisor™ HD Grid mapping catheter, Sensor Enabled™ (Abbott, Minneapolis, MN, USA) revealed recurrent conduction in the left and right superior PVs. The distal portions of both inferior PVs were electrically isolated, but the conduction resumed in the CT‐iPVs (Figure 2A–D; Movie S1). Furthermore, conduction of the LA posterior wall was also noted, with a little evidence of ablation of the LA roof and lower part of the LA posterior wall (Figure 2A–D). Because the anterior–posterior diameter of the left atrium was extremely small (30 mm) and the CT‐iPV ostium was not round (long/short diameter, 27/20 mm; Figure 1D), catheter manipulation and stabilization in the LA and balloon occlusion at the CT‐iPVs ostium were expected to be difficult. The CT‐iPVs protruded posteriorly, and the left inferior PV was proximal to the esophagus (Figure 1B,C). Therefore, pulsed‐field ablation (PFA), which allows precise catheter placement at the target site along a guidewire and does not require a complete occlusion of the PV for ablation, was performed using a circular catheter (PulseSelect™, Medtronic, Minneapolis, MN, USA). For the isolation of the CT‐iPVs, a guidewire was inserted into the distal portion of each inferior PV, and the PFA was delivered at the ostium of the CT‐iPVs. With 31 PFA applications delivered to the conduction resumption sites, the LA posterior wall, including the PVs, was completely electrically isolated (Figure 2E–H). No untoward events or recurrences were recorded during an 8‐month follow‐up without any antiarrhythmic drugs.

**FIGURE 1:**
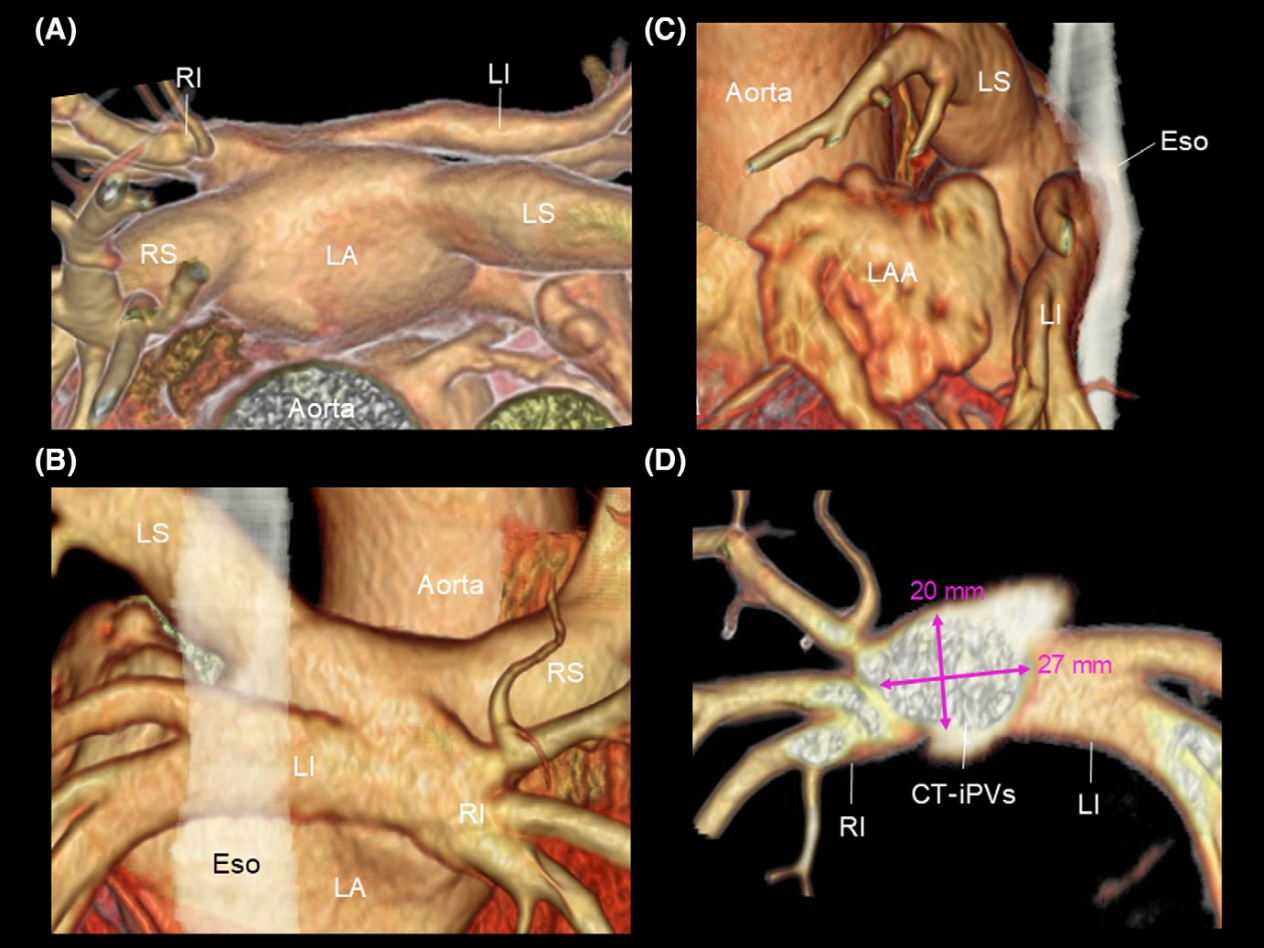
Three‐dimensional computed tomography reconstruction images before ablation. (A) Superior view, (B) posterior view, (C) left lateral view, (D) a cross‐section of the CT‐iPVs in the anterior view. The esophagus is indicated by a white translucent zone. CT‐iPVs, common trunk of inferior pulmonary veins; Eso, esophagus; LA, left atrium; LI (S), left inferior (superior) pulmonary vein; RI (S), right inferior (superior) pulmonary vein.

**FIGURE 2:**
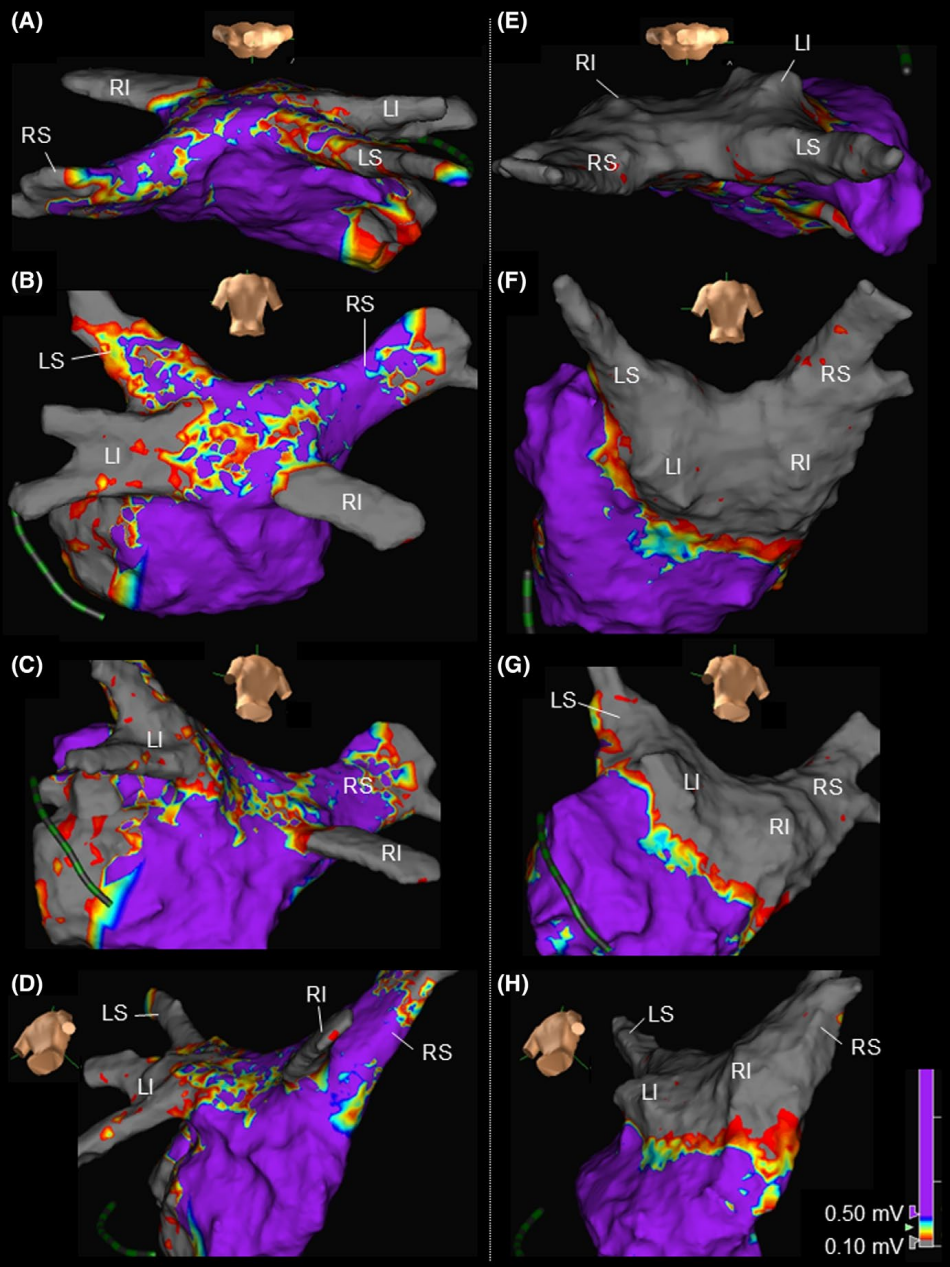
Ultra‐high‐resolution voltage mapping during coronary sinus pacing with an Advisor™ HD Grid mapping catheter, Sensor Enabled™ (Abbott, Minneapolis, MN, USA) before (A–D) and after ablation (E–H). Before the ablation, the conduction had resumed in the common trunk of the inferior pulmonary veins and left atrial (LA) posterior wall. Furthermore, low‐voltage areas, defined as less than 0.5 mV, were scattered on the LA roof and in the lower part of the posterior LA wall, which were considered to be traces of the radiofrequency catheter ablation at the previous hospital. The other abbreviations are as in Figure 1.

A CT‐iPV is a rare variant (0.9%–1.5%), and its entrance is often oddly shaped and distorted.^1^, ^2^ In PFA, it is important to keep the catheter in contact with or close to the target tissue; however, it is not necessary to press hard against the tissue or seal the PV, as with a cryoballoon.^3^ Furthermore, PV isolation with PFA has very few complications compared with radiofrequency ablation or cryoballoon ablation.^3^, ^4^ In this case, AF recurred after isolation of the PVs and LA posterior wall with radiofrequency energy. Despite the nonround ostium of the CT‐iPVs and extremely short anterior–posterior LA diameter, the PFA was able to reliably and safely isolate the CT‐iPVs. To our knowledge, this is the first report of the successful isolation of a CT‐iPVs using PFA, highlighting that PFA may be easier and more reliable than conventional ablation methods for PV isolation in patients with PV anomalies (unusual PV geometry) and/or a small LA.

Currently, an LA posterior wall isolation with PFA is an off‐label use in Japan. Recent studies have shown that a posterior LA wall isolation with PFA appears to be safe to perform, but the efficacy of performing an LA posterior wall isolation in addition to the PV isolation has not yet been established (Table S1). In this case, the patient had undergone radiofrequency catheter ablation of the LA posterior wall in addition to the PV isolation at another hospital (first session). In the voltage mapping, sporadic low‐voltage areas were observed on the LA roof and lower portion of the LA posterior wall (Figure 2C,D), which were thought to be traces of the LA posterior wall ablation during the first session. Therefore, to prevent the occurrence of atrial tachyarrhythmias involving the LA posterior wall as much as possible, we performed the LA posterior wall isolation with PFA.

## CONFLICT OF INTEREST STATEMENT

Drs. Machiko Miyoshi, Kanae Hasegawa, and Mr. Masato Shimada have nothing to disclose. Dr. Hiroshi Tada received honoraria for lectures or speaker bureaus from DAIICHI SANKYO COMPANY, Ltd., Novartis Pharma K.K., Medtronic Japan Co., Ltd., BIOTRONIK Japan, Inc., Bristol Myers Squibb, and Boston Scientific Japan K.K. Dr. Tada also received grants (Investigator initiated study unrelated to manuscript topic) from Abbott Medical Japan LLC, DAIICHI SANKYO COMPANY, Ltd., Nippon Boehringer Ingelheim Co., Ltd., Otsuka Pharmaceutical Co., Ltd., Eli Lilly Japan K.K., and Marubun Tsusyo K.K.

## ETHICS STATEMENT

The Research Ethics Committee of University of Fukui (20160040).

## CONSENT

Written informed consent was obtained.

## Supporting information


Table S1.

Movie S1.

## Data Availability

The data underlying the results are available within the article.
