# Supplementary material for: Pulsed‐field ablation to isolate common inferior pulmonary veins in a patient with recurrent atrial fibrillation
Source: J Arrhythm. 2025 Apr 9;41(2):e70058. doi: 10.1002/joa3.70058 (PMC11980092; doi:10.1002/joa3.70058)
Supplement: Supplementary file 1 — Table S1. [file JOA3-41-e70058-s002.docx]

**Supplementary Table-1. Recently-published articles of a left atrial posterior wall isolation using pulsed-field ablation**

| Authors | Journal, year | Subjects | Major results |
| --- | --- | --- | --- |
| Gunawardene MA, et al. | J Clin Med, 2023; 12: 6304. | 79 persistent AF pts: 59/79 pts were enrolled in the LAPWI cohort, including 16/59 index (27%) and 43/59 repeat ablation procedures (73%). 16 pts (16/79; 21%) were in the PVI-only cohort without LAPWI. | - LAPW isolation was performed successfully in all 59 (100%) pts of the LAPWI cohort. - Two minor complications occurred. No esophageal lesion was detected in the LAPWI cohort. - There was no difference regarding acute procedural and clinical outcome compared to the PVI-only cohort. - LAPWI guided by PFA is feasible and safe in patients undergoing catheter ablation for persistent AF and shows favorable outcomes. |
| Kordic LL, et al. | J Cardiovasc Electrophysiol, 2024;35:1525– 1535. | 94 pts (50%-persistent AF; 50%- long-standing persistent AF; 50%- 2^nd^ session. | - The acute ablation success rate was 100%. - Most favorable in persistent AF patients without extensive LA fibrosis. - The addition of LAPWI to PVI using multipolar PFA did not significantly influence procedure duration, transpired ablation time, or the rate of adverse events. |
| Badertscher P, et al. | J Intervent Cardiac Electrophysiol, 2024; 67: 1359- 1364. | 100 pts (24%-paroxysmal AF; 50%- long-standing persistent AF; 50%- 2^nd^ session. | - LAPWI using PFA was achieved in 100% of patients with a median of 19 applications (IQR 14–26). - No major complications. - In 15 pts (15%), recurrent AF/AT was noted during a median follow-up of 144 (94–279) days. |
| Turagam MK, et al. | JACC Clin Electrophysiol, 2024;10: 900- 912 | 547 pts with persistent AF: 131 (24%) received adjunctive LAPWI. | - 1-year freedom from atrial arrhythmias was not statistically different between groups in the full (PVI+LAPWI vs. PVI, P=0.68) and propensity matched cohorts (PVI+LAPWI vs. PVI: P=0.34). - No significant difference in major adverse events between the 2 groups - Adjunctive LAPWI did not improve freedom from atrial arrhythmia at 12 months. |
| Schiavone M, et al. | J Cardiovasc Electrophysiol, 2024; 35: 1101- 1111. | 249 pts: 21.7% had long‐standing persistent AF; LAPWI was performed in 57.6% of cases, with 15.3% being redo procedures. | - LAPWI was achieved in all patients by means of PFA alone, in 88.8% cases at first pass. - No major complications occurred. - During a median follow‐up of 273 days, no significant differences among ablation strategies (PVI+LAPWI vs. PVI). - LAPWI with PFA demonstrates feasibility, rapidity, and safety in real world practice, offering a viable alternative for persistent AF pts. |
| Kueffer T, et al. | Europace, 2024; 26: 1- 10 | 215 pts: 70.2%, persistent AF; 67.4%, redo ablation procedure. | - LAPWI was achieved in all pts by PFA. - Severe adverse events were cardiac tamponade and vascular access complication in one pt each (0.9%). - One-year arrhythmia-free outcome was 53%. - In 26 pts (12%) in whom redo procedure was performed, durable LAPWI was found in 22 pts (85%) with only minor lesion regression. Among 4 pts with LAPW reconnection, 3 (75%) presented with roof-dependent AT. - LAPWI by PFA can be safely and efficiently performed with a high durability observed during redo procedures. - The added value of durable LAPWI for the treatment of AF remains to be evaluated. |
| Pranata R, et al. | Heart Rhythm O2, 2024; 5: 720- 727 | Meta-analysis: 882 pts from 7 studies. | - The success rate of LAPWI was 100%. - No reported acute LAPW reconnection and esophageal complications. - No difference in terms of atrial tachyarrhythmia recurrence among LAPWI+PVI patients compared with those without LAPWI. - LAPWI may be considered during PFA, although the benefit is uncertain. |
| Casula, M et al | Int J Cardiol, 2025: 422: 132987. | Meta-analysis: 3,072 patients, (1,533- PVI and 1,539- LAPWI+PVI). | - According to our updated meta-analysis, the addition of LAPWI to PVI was not associated with a reduction of atrial arrhythmia recurrences. - A reduction of the risk of AF recurrence and a trend towards an increased risk of atrial flutter/AT were identified. |

Abbreviations: AF, atrial fibrillation; AT, atrial tachycardia; LA, left atrial; LAPW(I), left atrial posterior wall (isolation); pt(s), patient(s); PFA, pulsed-field ablation; PVI, pulmonary vein isolation.

**Movie S1.** Left atrial local activation time map with a sparkle propagation map during coronary sinus pacing before pulsed-field ablation using the Advisor™ HD Grid mapping catheter, Sensor Enabled™.
